# Reinforcement of Calcareous Sands by Stimulation of Native Microorganisms Induced Mineralization

**DOI:** 10.3390/ma16010251

**Published:** 2022-12-27

**Authors:** Gangqiang Shen, Shiyu Liu, Yuhan He, Muzhi Pan, Jin Yu, Yanyan Cai

**Affiliations:** 1 Fujian Research Center for Tunneling and Urban Underground Space Engineering, Huaqiao University, Xiamen 361021, China; 2 Fujian Water Conservancy and Hydropower Engineering Bureau Company Limited, Quanzhou 362000, China

**Keywords:** native microorganisms, stimulation solution, calcareous sand, response surface methodology, urease activity

## Abstract

Calcareous sand is a special soil formed by the accumulation of carbonate fragments. Its compressibility is caused by a high void ratio and breakable particles. Because of its high carbonate content and weak cementation, its load-bearing capacity is limited. In this study, the optimal stimulation solution was obtained with response surface methodology. Then, the effect of reinforcing calcareous sand was analysed with unconfined compressive strength (UCS) tests, calcium carbonate content tests, microscopy and microbial community analyses. The components and concentrations of the optimal stimulation solution were as follows: sodium acetate (38.00 mM), ammonium chloride (124.24 mM), yeast extract (0.46 g/L), urea (333 mM), and nickel chloride (0.01 mM), and the pH was 8.75. After the calcareous sand was treated with the optimal stimulation scheme, the urease activity was 6.1891 mM urea/min, the calcium carbonate production was 8.40%, and the UCS was 770 kPa, which constituted increases of 71.41%, 35.40%, and 83.33%, respectively, compared with the initial scheme. Scanning electron microscopy (SEM) and X-ray diffraction (XRD) analyses showed that calcium carbonate crystals were formed between the particles of the calcareous sand after the reaction, and the calcium carbonate crystals were mainly calcite. Urease-producing microorganisms became the dominant species in calcareous sand after treatment. This study showed that biostimulation-induced mineralization is feasible for reinforcing calcareous sand.

## 1. Introduction

Calcareous sand is a special geotechnical material with internal porosity, and it is easily broken into small particles [1]. Traditional reinforcement techniques use large amounts of cement, but the CO_2_ generated during cement production can exacerbate the problems caused by the greenhouse effect [2]. With the advocacy of green environmental protection, microbially induced carbonate precipitation (MICP) technology has been developed rapidly throughout the world. MICP technology uses urease-producing microorganisms to decompose urea and generate ammonium ions, which react with calcium ions to eventually generate calcium carbonate precipitates. The main chemical reactions of this process are shown in Equations (1)–(4) [3]:

(1) $$\mathrm{CO}{\left({\mathrm{NH}}_{2}\right)}_{2}+H_{2}O\overset{\mathrm{urease}}{\to }{\mathrm{NH}}_{3}+{\mathrm{NH}}_{2}\mathrm{COOH}$$

(2) $${\mathrm{NH}}_{2}\mathrm{COOH}+H_{2}O\to H_{2}{\mathrm{CO}}_{3}+{\mathrm{NH}}_{3}$$

(3) $$H_{2}{\mathrm{CO}}_{3}\to 2H^{+}+{\mathrm{CO}}_{3}^{2-}$$

(4) $${\mathrm{Ca}}^{2+}+{\mathrm{CO}}_{3}^{2-}\to {\mathrm{CaCO}}_{3}↓$$

Studies have shown that the calcium carbonate precipitate generated by the mineralization of MICP technology can not only improve the mechanical strength and liquefaction resistance of the material but also reduce the permeability [4,5,6,7,8]. At the same time, MICP technology can be applied to the process of oil production to prevent sand production [9]. It effectively reduces the system permeability and repairs the leaking pipeline and improves oil recovery [10,11]. However, the consolidation of geotechnical materials by introducing exogenous urease-producing microorganisms such as *Sporasarcina pasteurii* is limited by the bacterial culture and injection methods and may also result in the ecological problem of alien species invasion [12].

Therefore, the use of selective nutrients or environmental factors to stimulate native urease-producing microorganisms to strengthen geotechnical materials has become a popular topic of research in recent years [13,14,15]. Biostimulation can strengthen highly plastic clay, which can increase clay strength and reduce swelling [16]. Biostimulation has a certain reinforcement effect in deserts with low organic matter contents [17]. Biostimulatory effects can be boosted in places with high levels of organic carbon [18]. Biomineralization has been used to successfully improve the mechanical strength of eight different sands, and the unconfined compressive strength (UCS) results of the corresponding studies reached up to 5.3 MPa [19]. Soil treatments with biostimulation at different depths showed that, although the calcite content was reduced at locations farther away from the injection site, calcite precipitation was still induced at a depth of 12 m [20]. When the ability of different nutrients to stimulate native microorganisms was tested, it was concluded that yeast extract (YE) had a significant effect on activating native urease-producing microorganisms [21]. Biostimulation enables the enrichment of native urease-producing microorganisms with a simple carbon source (molasses), even in semiarid and nutrient-deficient environments [22]. A biostimulation solution successfully activated native urease-producing microorganisms in calcareous sands using YE, malt extract, nutrient broth, and urea; it supplied excess nutrients and added ammonium to increase the activation probability [23]. By comparing the mineralization effects of different stimulation solutions, it was shown that, within a certain range, the higher the content of YE was, the better the activation effect. At the same time, excessive nutrient content is not conducive to the uniformity of mineralization, and an appropriate concentration of nutrients can improve the overall reinforcement effect [12]. The choice of stimulation solution has an important influence on the reinforcement effect, and a reasonable stimulation scheme can lead to a better reinforcement effect and save time and cost. However, there is little relevant research on optimizing stimulation solutions.

In this paper, a biostimulation scheme based on response surface methodology optimization is proposed, and relevant experiments are carried out using calcareous sand. The evaluation of the effect of reinforcing calcareous sand with this scheme reveals its reinforcement mechanism and influencing factors. This study provides a new method and basis for the optimization of biostimulation programs and the foundation treatment of calcareous sand.

## 2. Materials and Methods

### 2.1. Materials

Starch, corn steep liquor and soybean meal were purchased from Beijing Hongrun Baoshun Technology Co., Ltd. (Beijing, China), and molasses was purchased from Guangxi Jianli Chemical Trading Co., Ltd. (Nanning, China). All other reagents were of analytical grade and were purchased from China National Pharmaceutical Group Corporation. Deionized water was used as the configuration solution in the tests.

The material sample used in the test is calcareous sand taken from an island reef in the South China Sea. Calcareous sand is mainly composed of calcium carbonate, which is porous and irregular in shape, with a small amount of shell debris. According to the specifications of soil testing [24], the particle sizes were mainly concentrated at approximately 0.5–1.0 mm, and the particle parameters of calcareous sand, which is classified as poorly graded, are shown in Table 1.

### 2.2. Biostimulation

Using previous research on biostimulation reinforcement materials and methods [9,14,17,25], the species of the substances used for the stimulation scheme were identified, and calcareous sand was used in the tests. The concentrations of substances in the initial stimulation solution were set as sodium acetate (42.5 mM), ammonium chloride (100 mM), urea (333 mM), YE (0.2 g/L), and nickel chloride (0.01 mM), and the pH was 7.0. The test was performed as follows: First, 90 mL of stimulation solution was added to a 300 mL conical flask. Then, 10 g of well-mixed and air-dried calcareous sand sample was added. Finally, the mixture was shaken well in a conical flask and cultured in a shaking incubator at a constant 30 °C and 180 rpm. The optimal stimulation solution of calcareous sand was obtained through a single-factor test, a Plackett-Burman (PB) test, and the response surface analysis method was the central composite design (CCD) method; environmental factors such as pH and urease activity were used as the evaluation criteria.

#### 2.2.1. Single-Factor Test

Different kinds of nutrients in the microbial growth phase have a great influence. To biostimulate native microorganisms that can reach the highest urease activity, the single-factor test was based on the initial stimulation solution. Only the type of carbon source or nitrogen source in the stimulation solution was changed, and the urease activity in the reaction system was evaluated, thus selecting the best carbon source and nitrogen source.

In the single-factor carbon source test, sodium acetate in the initial stimulation solution was replaced with monosaccharides (glucose, fructose), disaccharides (maltose), polysaccharides (starch) and sugar by-products (molasses), and other parameters were kept unchanged. Three parallel samples were used in each group. Urease activity was measured at 0, 6, 12, 24, 48, 72, and 96 h in a conical flask. Electrical conductivity (EC) was measured using a Con700 conductivity meter produced by Eutech Instruments Pte Ltd., Singapore. Urease activity (UA) was the change in the conductivity of a 3 mL sample and 27 mL 1.5 mol/L urea mixed solution within 1–9 min, which was calculated from Equation (5) [26]:

(5) $$\mathrm{UA}=\frac{{\mathrm{EC}}_{9\min}-{\mathrm{EC}}_{1\min}}{8}\times 10\times 11.11$$

Among them, the units of UA are mM urea/min, the coefficient of 10 is used as the dilution factor of the sample, and the coefficient of 11.11 represents the relationship between the change in EC and the concentration of hydrolysed urea.

The nitrogen source single-factor test was similar to the carbon source single-factor test. Different nitrogen sources were used in each group, either an organic nitrogen source (corn steep liquor, soybean meal, or starch) or an inorganic nitrogen source (ammonium sulfate, ammonium chloride, or sodium nitrate). An organic nitrogen source and inorganic nitrogen source were selected because microorganisms use them at different stages of growth. An organic nitrogen source is a low-efficiency nitrogen source that has low utilization in the early stage but can increase the number of microorganisms in the late stage. However, inorganic nitrogen is a high-efficiency nitrogen source that is quickly used and grown by microorganisms. These nitrogen sources meet the growth requirements of most native microorganisms in calcareous sands. The specific steps are the same as those in the optimal carbon source test.

#### 2.2.2. PB Test Design and Screening of Key Components

The PB test is a two-level screening test that can compare the key influencing factors that have the most significant effect on the test results [27]. In the single-factor test results, two substances with high urease activity were selected from the carbon source, organic nitrogen source, and inorganic nitrogen source for the PB test. The urea, YE, nickel chloride, and pH in the initial stimulation solution were also studied as key influencing factors. Considering the above factors, with urease activity as the target value, significant influencing factors were determined through tests designed with Minitab 16 software.

#### 2.2.3. CCD Response Surface Analysis

CCD can be used to find the optimal key influence parameters by fitting the functional relationship between each factor and the target response value when only partial data are obtained. The key factors selected by the PB test were subjected to a single-factor test to obtain the most reasonable concentration range. After the concentration of key factors was determined, the CCD test determined with Minitab 16 software was used to obtain the optimal stimulation solution suitable for calcareous sand.

### 2.3. Biomineralization Scheme

#### 2.3.1. Sample Preparation

All moulds and grouting devices were sterilized before the test. The mould was assembled from polyvinyl chloride (PVC) tubes (8.0 cm in length, 3.5 cm in inner diameter), two rubber stoppers, and a fixed bracket. When preparing the sample, Vaseline was first applied to the PVC tube. Then, layered filling of calcareous sand was used to maintain the uniformity of the sand column. Finally, the amount of calcareous sand in each sand column was controlled to 100 ± 1 g. Both ends of the sand column were padded with nylon filters to prevent particle loss during grouting. After filling, a plastic hose was used to connect with the peristaltic pump, and the plastic hose was made without air circulation before the test started to reduce the influence of miscellaneous bacteria.

#### 2.3.2. Biomineralization Scheme and Parameter Setting

**Figure 1:**
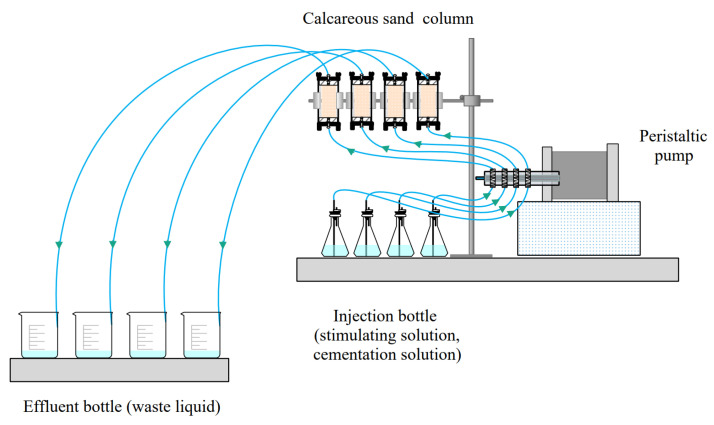
Schematic diagram for the grouting process.

**Figure 2:**
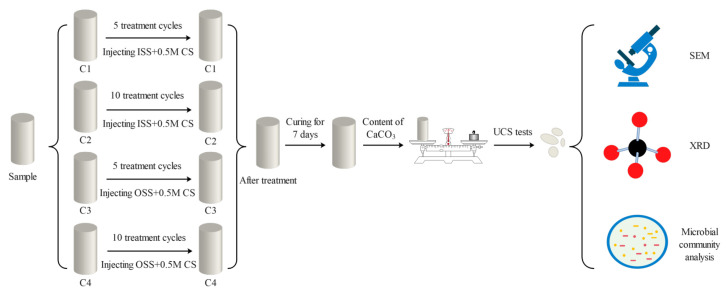
Mineralization scheme and parameter setting (ISS, initial stimulation solution; OSS, optimized stimulation solution; CS, cementation solution).

The mineralization test device is shown in Figure 1. Grouting includes two stages of activation and mineralization, both of which adopt the grouting method from bottom to top. The whole test was carried out at 30 °C. In the activation stage, 50 mL of stimulation solution was injected into the sand column and incubated for 72 h to activate the urease-producing microorganisms. In the mineralization stage, first, 50 mL of stimulation solution was injected, and then an equal amount of cementation solution was injected; the cementation solution was a mixed solution of 50 mL of equimolar calcium chloride and urea. The interval between each mineralization was 24 h to ensure a complete mineralization reaction. Biomineralization parameters were set for different treatment cycles, including 5 and 10 cycles, and 0.5 mol/L calcium chloride was selected [7,28]. The control group was mineralized with the initial stimulation solution, which was used to compare the difference in the reinforcement effect of the optimal stimulation solution. The specific scheme is given in Figure 2. The tests were divided into 4 groups, and each group had 3 parallel samples.

### 2.4. Evaluation of the Biomineralization Effect

The initial stimulation solution and the optimal stimulation solution were used to reinforce the calcareous sand. Urease activity, calcium carbonate content, UCS, scanning electron microscopy (SEM), X-ray diffraction (XRD), and microbial community analysis were used to evaluate the mineralization effect.

#### 2.4.1. Urease Activity

Urease decomposes urea with high specificity. High urease activity can decompose more urea per unit time; therefore, more calcium carbonate precipitates can be produced during the test, which improves the reinforcement. Under the same conditions, the urease activity of the calcareous sand treated with the initial and optimal stimulation solutions was measured. The high efficiency of the optimal stimulation solution was verified.

#### 2.4.2. Determination of Calcium Carbonate Content

After the mineralization test, the sand column was left standing for 3 days before washing, drying, and demoulding. The weight of the sand column that was dried to a constant weight in an oven at 60 °C was recorded as M_2_, and the weight of the sand column before preparation was recorded as M_1_. The calculation formula of calcium carbonate content (C) is shown in Formula (6). The calcium carbonate content is an important reference index used to measure the reinforcement effect. However, the composition of the calcareous sand itself is mainly calcium carbonate, so the content of calcium carbonate cannot be determined by methods such as the pickling method or titration method. Therefore, it can only be calculated according to the change in the amount of calcareous sand in a column from before mineralization to after mineralization.

(6) $$C=\frac{M_{2}-M_{1}}{M_{1}}\times 100\%$$

#### 2.4.3. UCS Test

The dried calcareous sand column was subjected to UCS testing. The loading rate was 1 mm/min until the specimen failed. The instrument used was a TSZ-3 strain control triaxial apparatus from Nanjing Soil Instrument Factory Co., Ltd., Nanjing, China.

#### 2.4.4. SEM and XRD Analysis

After drying and spraying gold, the sand column samples were tested by SEM to observe their microscopic morphology and structure. The SEM test equipment adopted a Sigma 300 field emission scanning electron microscope produced by Zeiss, Germany, and the test acceleration voltage was 10 or 15 kV. Samples were taken for XRD testing to compare and analyse the change in material composition in calcareous sand before and after biostimulated mineralization. The equipment used in the XRD test was a SmartLab produced by Rigaku Corporation, Japan. The working voltage of the sample test was 40 kV, the current was 30 mA, the angle parameters of the XRD test were set to 5~60°, and the step size was 0.02.

#### 2.4.5. Microbial Community Analysis

Sand samples were taken for microbial community analysis. Total deoxyribonucleic acid (DNA) was extracted from calcareous sand and subjected to polymerase chain reaction (PCR) amplification and sequencing using OMEGA’s E.Z.N.A.™ Mag-Bind Soil DNA Kit for DNA extraction. The final process of high-throughput sequencing was performed according to the Illumina16S ROD database sequencing library preparation guidelines of Shanghai Sangon Biotechnology Company. Based on the results, the diversity and abundance of microbial communities in the samples before and after biostimulatory mineralisation treatments were evaluated.

## 3. Results and Discussion

### 3.1. Optimal Stimulation

#### 3.1.1. Single-Factor Test

**Figure 3:**
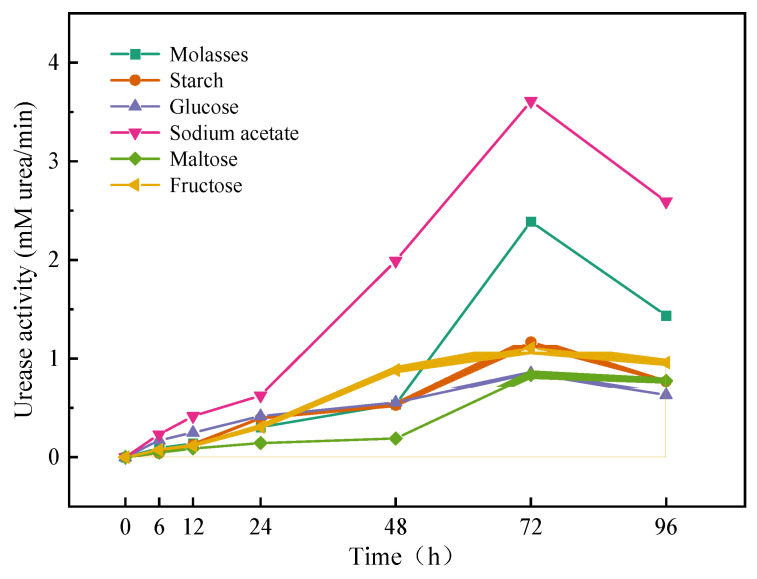
Effect of different carbon sources on urease activity of samples (shadows represent the range of error).

In the single-factor test of carbon sources, the urease activity of the different carbon sources changed with time, as shown in Figure 3. The urease activity increased continuously from 0 to 72 h and reached a maximum at 72 h. The urease activity changed the most between 48 and 72 h, indicating that urease-producing microorganisms had the highest activity during this period and produced more ions by decomposing urea. After 72 h, the urease activity decreased. This may be because the nutrients in the reaction system were almost completely consumed, and the microorganisms could not grow and produce urease to decompose the urea. At the same time, part of the previous urease might have been inactivated. The results showed that the urease activity was highest in the two groups that used sodium acetate and molasses as carbon sources, so these two carbon sources were considered the optimal carbon sources.

**Figure 4:**
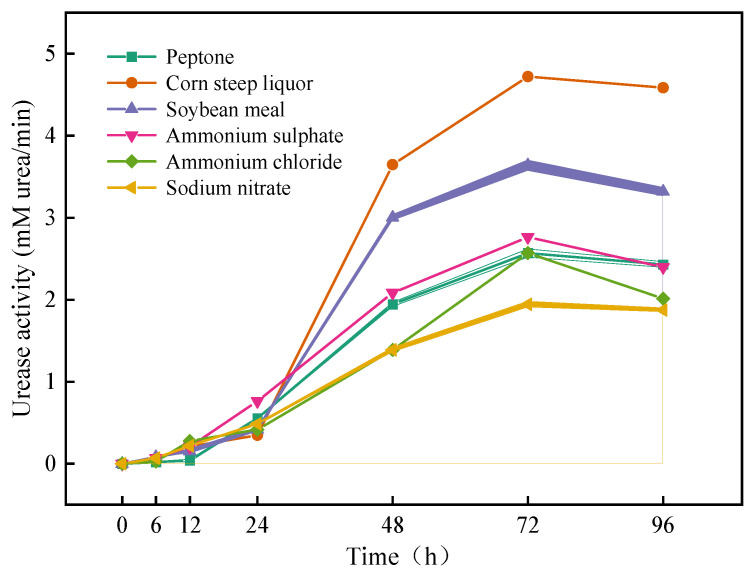
Effects of different nitrogen sources on the urease activity of samples (shadows represent the range of error).

In the single-factor test of nitrogen sources, the urease activity of the different nitrogen sources changed with time, as shown in Figure 4. Similar to the carbon source test, the urease activity also reached a maximum at 72 h and then decreased. The urease activity of the organic nitrogen source group was higher than that of the inorganic nitrogen source group. When soybean meal and corn steep liquor were used as nitrogen sources, the urease activities were the highest. However, soybean meal and corn steep liquor are both organic nitrogen sources, and they led to high urease activity because of the influence of compound nitrogen sources. Later, the organic nitrogen source and the inorganic nitrogen source will be used in combination to further determine the optimal nitrogen source.

#### 3.1.2. Results of PB Design

According to the single-factor test results, two factors (molasses, sodium acetate, corn steep liquor, soybean meal, ammonium sulfate, ammonium chloride) with the highest urease activity were selected from the carbon sources, organic nitrogen sources, and inorganic nitrogen sources for the PB tests. Urea, YE, nickel chloride, and pH were selected as the key influencing factors.

The PB test with N = 12 test times was performed with urease activity as the target value. The factors and levels of the experimental design are shown in Table 2, the results of the scheme design are shown in Table 3, and the analytical results are shown in Table 4. The equation obtained by fitting the data was: Y = 2.8067 + 0.0083A − 0.2617B + 0.0167C − 0.2017D + 0.0500E + 0.6300F − 0.1317G − 0.4083H + 0.3983I + 1.3000J. The factors A, B, C, D, E, F, G, H, I, and J represent molasses, sodium acetate, corn steep liquor, soybean meal, ammonium sulfate, ammonium chloride, nickel chloride, urea, YE, and pH, respectively. The *P* value of the model was less than 0.05, which shows that the model had a significant influence. The determination coefficient R^2^ for the model was greater than 99%, indicating that 99% of the data in this model were explained by this model. The results showed that the p values of pH, ammonium chloride, urea, YE, and sodium acetate were all less than 0.05, indicating that those parameters significantly affected urease activity. The *p* value of the model was also less than 0.05, indicating that the model had a significant influence. The components of the stimulation solution were divided into four parts: carbon source, nitrogen source, growth factor, and environmental factor. According to the above analysis, sodium acetate, ammonium chloride, YE, and pH were selected from these four parts as the key factors affecting urease activity.

#### 3.1.3. CCD Response Surface Analysis

**Figure 5:**
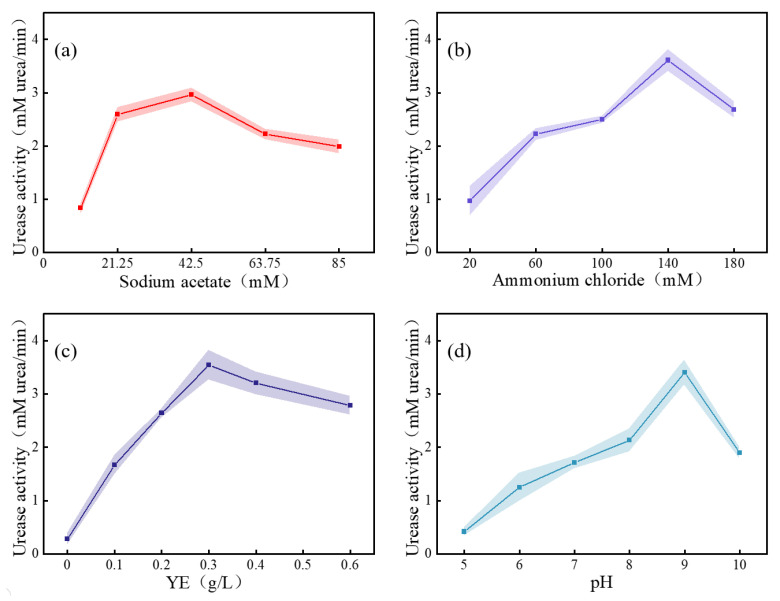
Effect of each key factor on urease activity: (**a**) sodium acetate; (**b**) ammonium chloride; (**c**) YE; and (**d**) pH.

The key factors obtained from the PB test were subjected to a single-factor test to explore the most reasonable range of addition. According to the positive and negative effects of various factors on urease activity shown in Table 4, the stimulation solution was adjusted as follows: sodium acetate (42.50 mM), ammonium chloride (151 mM), YE (0.3 g/L), urea (333 mM), nickel chloride (0.01 mM), and the pH value was 9. Figure 5 shows the effect of the concentration of each key factor on urease activity. The urease activity reached a maximum when the concentrations of sodium acetate, ammonium chloride, and YE ranged from 21.25 mM to 63.75 mM, 100 mM to 180 mM, and 0.2 g/L to 0.4 g/L, respectively, and the pH ranged from 8 to 10.

Minitab 16 software was used for the design and analysis of the CCD test with four factors and five levels. The concentrations of four influencing factors, sodium acetate, ammonium chloride, YE, and pH, were used as independent variables, and urease activity was used as the response value. The levels of each factor are shown in Table 5, and the other components of the stimulation solution were urea (333 mM) and nickel chloride (0.01 mM). The analysis of variance in the model results is shown in Table 6. The *p* < 0.0001 of the regression model and the *p* = 0.244 of the misfit term are greater than 0.05, indicating the model and test data can be fitted accurately; the *R*^2^ value was 95.39%, and the *R*^2^ (adj.) value was 91.35%, indicating that the prediction results of the model were reliable.

**Figure 6:**
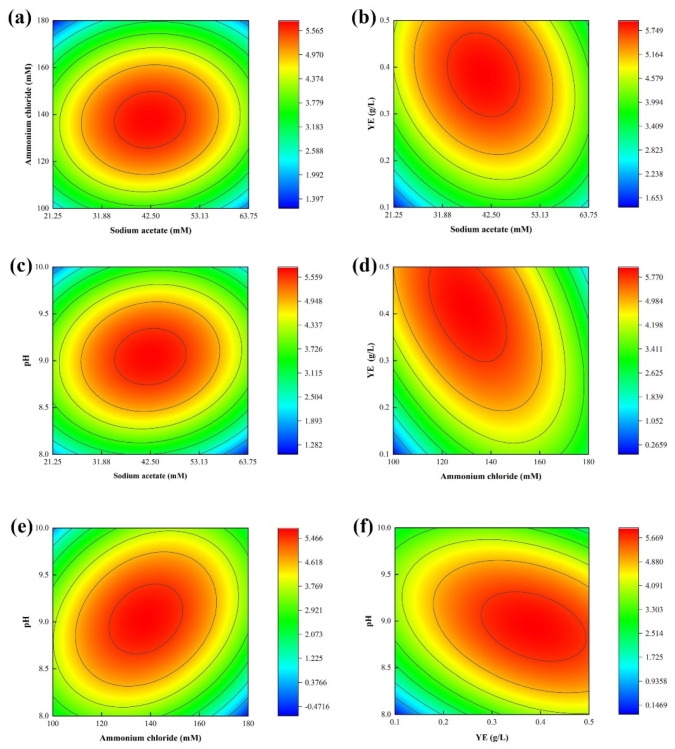
Contour plots of urease activity under the influence of two factors: (**a**) sodium acetate and ammonium chloride; (**b**) sodium acetate and YE; (**c**) sodium acetate and pH; (**d**) ammonium chloride and YE; (**e**) ammonium chloride and pH; (**f**) YE and pH.

Figure 6 shows the contour curves for changes in urease activity resulting when the influencing factors were combined in pairs. To indicate the levels of urease activity in the different regions, different colours were used to mark the ranges of urease activity. The deepest red in the graphic indicates the maximum urease activity possible under the current conditions. Wider intervals between the contours corresponding to a factor indicate it had less influence on urease activity, and the different influencing factors had different effects on urease activity. Therefore, urease activity only maintained a high level within the optimal ranges of the key factors. When its concentration was too high or too low, the urease activity decreased. As can be seen from the figure, the urease activity obtained by fitting the data exceeded 5.5 mM urea/min, which is much higher than the activity obtained with the initial stimulation scheme. In the ranges of the concentrations for the various factors, pH and ammonium chloride had the greatest effects on urease activity. This is also consistent with the fact that these are the two most significant factors influencing the PB test. pH has a great effect on the growth and urease activity of microorganisms [9,29,30]. Ammonium chloride is a nitrogen source and stimulates the growth of ammonia-oxidizing microorganisms [22]. They have great impacts on the growth of microorganisms.

**Figure 7:**
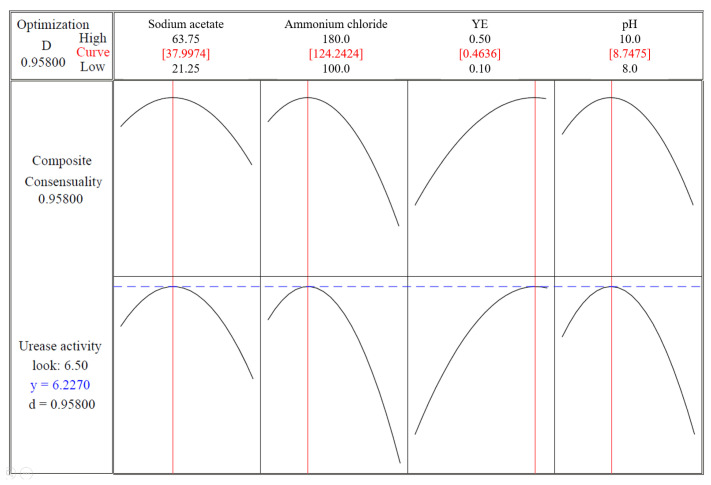
CCD test optimization curve.

Figure 7 shows the optimization curve of the CCD test. The optimal fitting value of the urease activity was 6.2270 mM urea/min, and the concentrations of the components in the stimulation solution were as follows: sodium acetate (38.00 mM), ammonium chloride (124.24 mM), YE (0.46 g/L), urea (333 mM), and NiCl_2_ (0.01 mM); the pH value was 8.75.

### 3.2. Urease Activity

The average urease activity of calcareous sand treated with the optimal stimulation solution was 6.1891 mM urea/min, which was consistent with the fitting value and represented an increase of 71.41% when compared with that of the initial stimulation solution. The main reason for the improvement in urease activity was that the optimal stimulation solution was compatible with more urease-producing microorganisms when activating native microorganisms. Therefore, more urea could be decomposed during the test, making the electrical conductivity rise quickly. It is generally believed that the higher the urease activity is, the faster the rate of decomposition of the urea, which can reduce the time and cost requirements of biomineralization [31].

### 3.3. Calcium Carbonate Content

**Figure 8:**
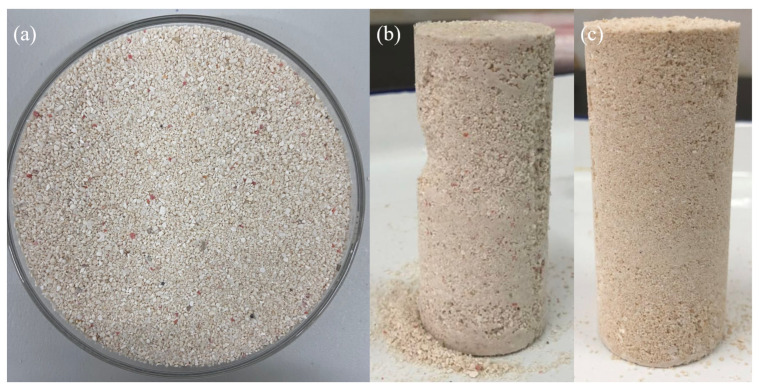
Comparison of calcareous sand before and after biomineralization: (**a**) untreated calcareous sand; (**b**) and (**c**) calcareous sand columns formed by Groups C2 and C4, respectively.

**Figure 9:**
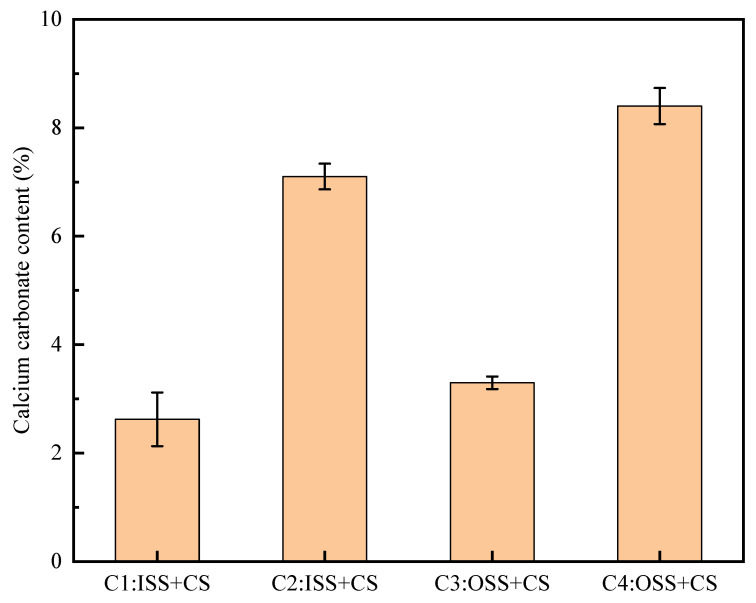
Calcium carbonate contents of samples.

Figure 8 shows a comparison of calcareous sand before and after biomineralization. Calcareous sand was stably formed after 10 cycles of biomineralization. The calcium carbonate produced by biomineralization strengthens calcareous sand by filling the pores and increasing the contact area of the particles [32]. However, the appearance of the sand column in Group C2 is not as smooth as that in Group C4, and some sand columns fall apart when the sample is demoulded. It may be that some locations are not mineralized enough to make the sand column more densely packed. The calcium carbonate content in each group measured by the weighing method is shown in Figure 9. The calcium carbonate content of calcareous sand column treated with the initial stimulation scheme was lower than that treated with the optimal stimulation scheme. The average content of calcium carbonate in the sand column of Group C2 was only 2.62%. This may be the reason why the initial stimulus method did not activate more urease-producing microorganisms. However, the number of mineralization rounds was doubled, and the content of calcium carbonate was not worth doubling. For any given stimulation solution, more treatment cycles resulted in more generation of calcium carbonate. After 10 cycles of mineralization, the calcium carbonate content of the sand column was between 6% and 9%. The average calcium carbonate content of the calcareous sand column in Group C4 was 8.40%, which was 35.40% higher than that in Group C2. Previous studies have shown that when the calcium carbonate content is greater than 5%, the strength of the sample is effectively improved and can meet the requirements of most engineering applications [33,34,35]. Moreover, the higher the urease activity is, the higher the calcium carbonate content. This is because the higher urease activity scheme can produce more carbonate acid at the same time, accelerating the rate of mineralization [36].

### 3.4. UCS Test and Analysis

**Figure 10:**
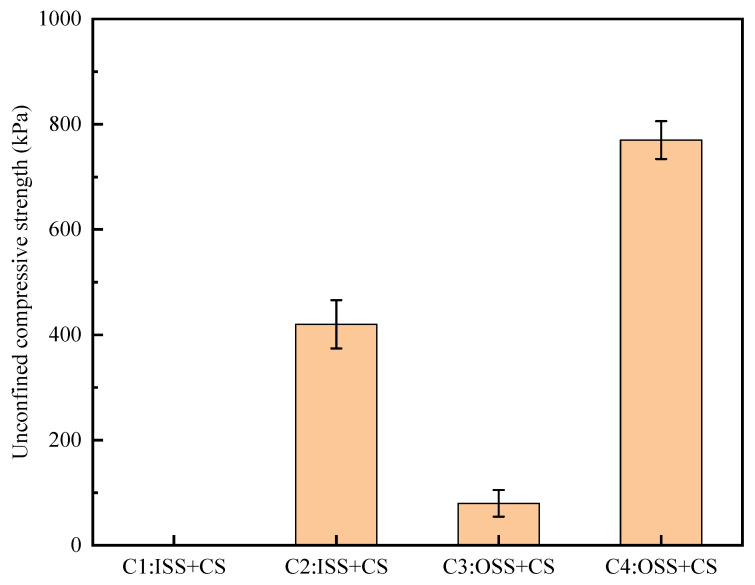
Uniaxial compressive strengths of the samples.

The UCS results of the calcareous sand columns in each group are shown in Figure 10. Group C1 fractured during demoulding due to a short mineralization time, so the UCS was 0. The content of calcium carbonate was lowest in Group C2. However, the strengths of the samples treated with the optimal stimulation scheme were improved, and the unconfined compressive strengths reached 80 kPa. This increase also occurred in the 10-round test scheme of mineralization, and the UCS of Group C4 reached 770 kPa, which was 83.33% higher than that of Group C2. These results also showed that for a given stimulation solution, increasing the number of mineralization cycles significantly improved the mechanical strength of the sand column. The test shows that increasing the number of mineralization cycles and using the stimulation program with high urease activity can effectively improve the reinforcement effect.

**Figure 11:**
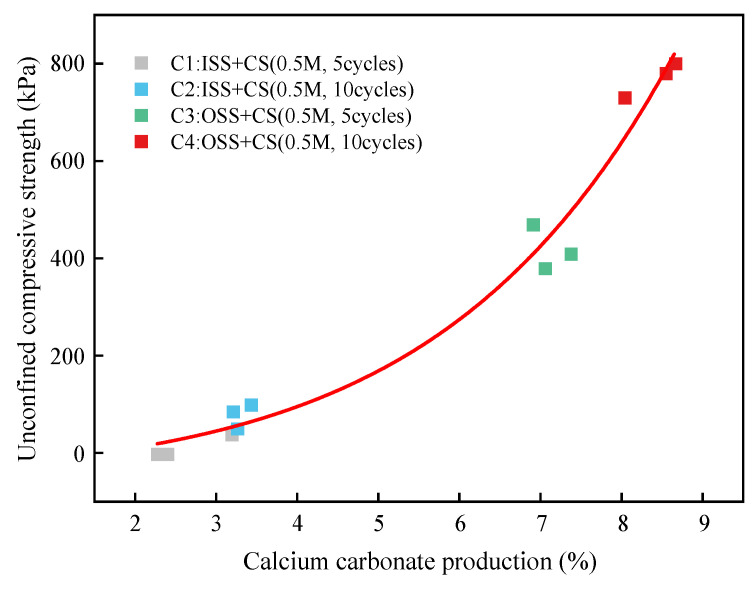
Relationship between calcium carbonate content and uniaxial compressive strength.

Figure 11 shows the relationship between the UCS and the calcium carbonate content of the sand column; there is clearly a positive correlation between the changes in the two. Increases in the calcium carbonate content did not effectively improve the strength of the sample when the content was less than 5%. In order to improve the strength of the sample significantly, the content of calcium carbonate in the sample was increased to more than 5%. Related studies have shown that when the content of calcium carbonate was higher than 10%, it effectively improved the mechanical strengths of the samples [37,38]. The higher the calcium carbonate content was, the greater the UCS, which agrees with the results of previous studies [12,39,40,41]. The results showed that the main reason for the increased mechanical strength of the sand column was the precipitation of calcium carbonate during the process of biomineralization. This is because the generated calcium carbonate can not only cement the calcareous sand particles and improve the contact point of the particles but can also improve the pores in the particles [42].

**Figure 12:**
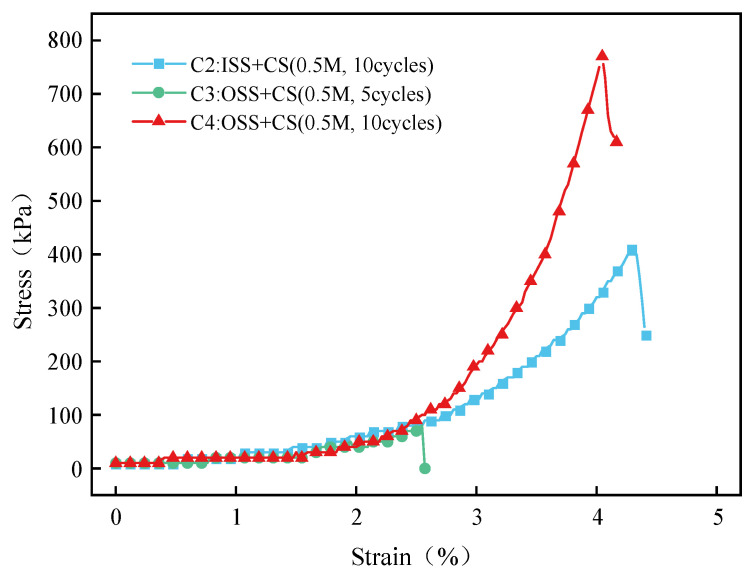
Stress-strain curve of the treated samples.

The stress-strain curves of each sand column are shown in Figure 12. The stress-strain curves of different groups of sand columns are quite different, indicating that the mineralization parameters have a great influence on the reinforcement effect of the sand column. However, with the increase in stress, the sand column goes through a compaction stage and an elastic stage. When the stress reaches the peak value, the sand column breaks, and the stress drops sharply. Compared with Group C4, the sand column in Group C2 has a large strain, a small stress peak value, and a slowly evolving elastic stage. This is likely due to the low calcium carbonate content of Group C2, which leads to poor cementation between sand particles [43].

### 3.5. SEM Test Results and Analysis

**Figure 13:**
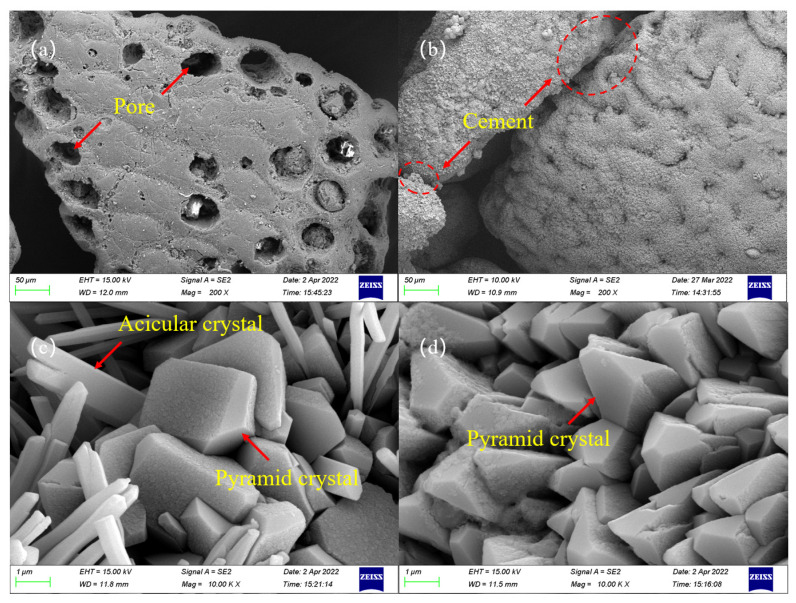
SEM of samples: (**a**–**d**) are the results of untreated sand, C2, C2, and G4 samples, respectively.

Figure 13 shows the SEM test results of some samples before and after calcareous sand mineralization. Figure 13a shows the SEM image of the untreated calcareous sand; the surface of the calcareous sand is uneven and distributed with many pores, which reflects the structure of the calcareous sand. Figure 13b shows the SEM image of calcareous sand in Group C2; the surfaces of the calcareous sand particles are covered by mineralization products filled with pores, and the particles are cemented with each other, which is the main reason for the increase in sample strength after mineralization. Figure 13c,d show the SEM test results of Groups C2 and C4 at higher magnifications, respectively. The morphology of the mineralization products differed between the two groups. Group C2 treated with the initial stimulation solution produced both needle-shaped and pyramid-shaped mineralization products, in which the needle-shaped crystals were increasingly smaller in size. However, the mineralization products of Group C4 treated with the optimal stimulation solution were all pyramid-shaped, and the crystal size was generally larger than that in Group C2. The difference in crystal shape between the two groups was mainly caused by the different stimulation solutions. It is generally believed that the crystal sizes of mineralization products are larger under high nutrition conditions, while small crystals dominate under low nutrition conditions [18]. The test results showed that the optimized stimulation solution improves the availability of nutrients, improves the nutritional conditions of urease-producing microorganisms, and is conducive to the formation of large crystals.

### 3.6. XRD Results and Analysis

**Figure 14:**
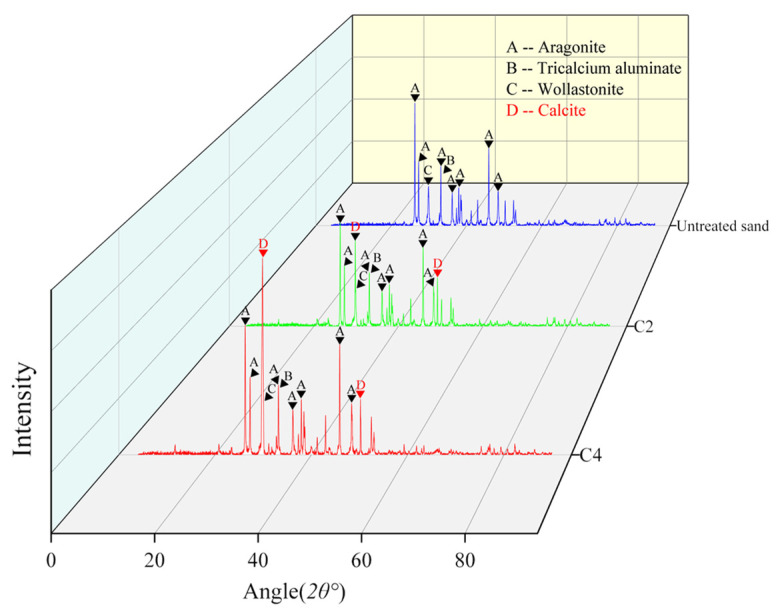
XRD analysis results of samples.

XRD can be used to analyse the crystal type of a sample. Calcium carbonate has various crystal types, mainly spheraragonite, calcite and aragonite, among which calcite is a relatively stable crystal type [7]. Figure 14 shows the XRD comparison of calcareous sand before and after mineralization. The main components of the calcareous sand are aragonite, tricalcium aluminate, and wollastonite, of which aragonite has the highest content. The absorption peaks of calcite appear in the XRD analysis of Groups C2 and C4 [44], indicating that the calcium carbonate generated during the mineralization process is mainly calcite. However, the intensity of the absorption peak of Group C4 is much higher than that of Group C2, indicating that Group C4 produces more calcite. The formation of calcite is the main reason for the increase in the strength of the calcareous sand [45]. Previous studies have also shown that calcite is the main crystal form of calcium carbonate produced by the reinforcement of sand by native microorganisms [46].

### 3.7. Results of Microbial Community Analysis

To study the changes in microbial communities within biostimulated, mineralized calcareous sands, the calcareous sand samples before and after mineralization were subjected to high-throughput sequencing analysis for comparison and explanation.

#### 3.7.1. Rank Abundance Curve

**Figure 15:**
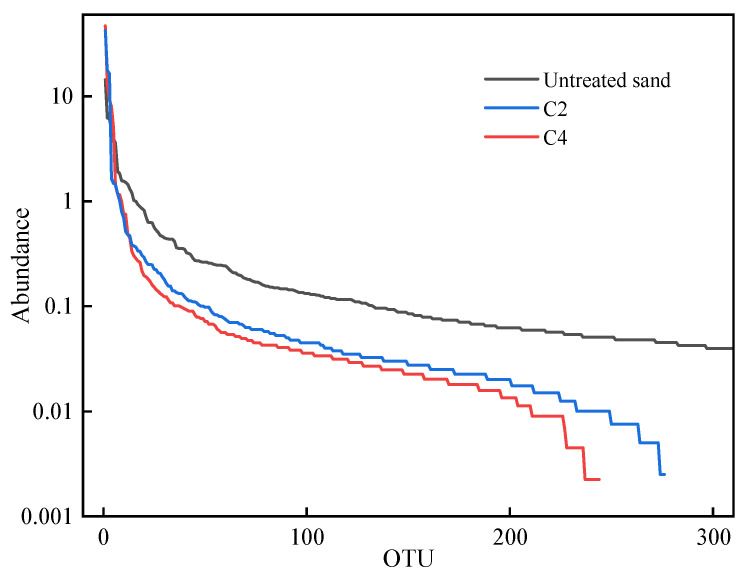
Rank abundance curves for different samples.

The rank abundance curves of Groups C2 and C4 and untreated calcareous sand are shown in Figure 15. The rank abundance curve can be used to explain two aspects of the sample diversity simultaneously, i.e., the richness and uniformity of the species contained in the sample. The richness of the species is reflected by the length of the curve along the horizontal axis, and the wider the curve, the more OUT grades it contains and the richer the composition of the species. The uniformity of species composition is reflected by the shape of the curve, and the flatter the curve is, the higher the uniformity of the species composition. The rank abundance curve of the untreated calcareous sand was flat, indicating the highest degree of uniformity of species composition. The rank abundance curve of the stimulated calcareous sand showed a step-like shape, indicating that part of the OTU group was missing. The rank abundance curve of Group C4 was most obvious, probably because those microorganisms could not adapt to the high-pH environment in the calcareous sand. The results showed that some native microorganisms could not survive normally due to a lack of nutrients after treatment with the stimulation scheme, but that nearly all microorganisms that used urea as a nitrogen source could survive normally. After the biostimulation treatment, the abundance and uniformity of the species in the sand were reduced, and the native urease-producing microorganisms were successfully activated and enriched.

#### 3.7.2. Microbial Relative Abundance Analysis

**Figure 16:**
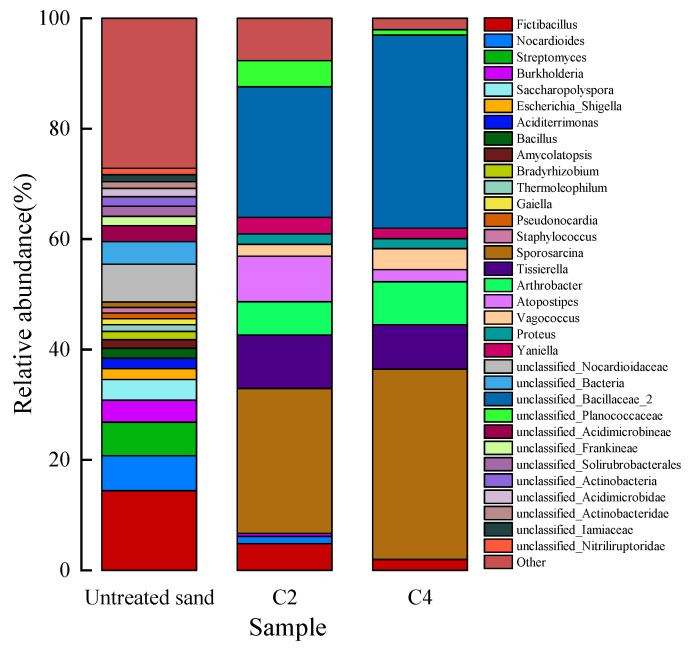
Relative abundance of microorganisms in different samples.

The community-level relative abundance changes (genus) of the mineralized calcareous sand compared to the untreated calcareous sand are shown in Figure 16. Different colours represent different bacteria, and the area of the graph represents the percentage of abundance. The results showed that the proportion of bacteria in calcareous sand changed obviously after the stimulation treatment. In the untreated calcareous sand, Fictibacillus (14.42%) and Nocardioides (13.18%) were the most common, followed by Streptomyces (6.08%) and Bacillus (5.88%). Firmicutes (Fictibacillus and Bacillus) have a remarkable ability to decompose urea [47]. Nocardioidaceae and Streptomyces contain some bacteria that can decompose urea and play an important role in the production of calcite [48,49]. The presence of these bacteria in untreated calcareous sand was the key to successful biomineralization. However, the abundance and quantity of Groups C2 and C4 were not significantly different, which may be the reason for using the same nutrient species in the stimulation scheme. They all have a common dominant strain (Sporosarcina and Bacillaceae). The results of Group C4 showed that Sporosarcina (34.47%) and Bacillaceae (34.98%) became the absolute dominant species, and the vast majority of Sporosarcina had the ability to produce urease. The proportions of these two bacteria in Group C4 were 6.13% and 9.44% higher than those in Group C2, respectively, indicating that the proportions of the microorganisms could be changed indirectly by changing the concentrations of the nutrients. The dominant species in the treated calcareous sand effectively decomposed urea, which promoted the success of biomineralization.

## 4. Conclusions

In this paper, the optimal biostimulation solution of calcareous sand was obtained by response surface methodology. The reinforcement effects before and after solution optimization were evaluated and compared, and the reinforcement mechanism was explained. The main conclusions are as follows:

1. Using response surface methodology, stimulation solutions were optimized based on the urease activity of indigenous microorganisms in calcareous sand. The following components and concentrations were obtained for the optimal stimulation solution: sodium acetate (38.00 mM), ammonium chloride (124.24 mM), yeast extract (0.46 g/L), urea (333 mM), and nickel chloride (0.01 mM); the pH value was 8.75.
2. The comparison of test results showed that the urease activity reached 6.2270 mM urea/min with the optimal stimulation solution. In the actual test, the urease activity reached 6.1891 mM urea/min, which was 71% higher than that of the initial stimulation solution. In mineralization tests, calcareous sand columns treated with the optimal stimulation regimen showed increased calcium carbonate precipitation. The high urease activity mineralization scheme and multiple cycles of treatment could effectively improve the UCS. The UCS of the calcareous sand column treated by the optimal stimulation scheme was significantly higher than that of the initial stimulation scheme, and the maximum reached 770 kPa.
3. The SEM and XRD analysis results showed that mineralization products formed between the calcareous sand particles and on their surfaces. Calcium carbonate obtained from biomineralization precipitated as calcite. The appearance of calcite and cementation between particles greatly improved the mechanical properties of calcareous sand.
4. Microbial community analysis showed that the stimulation scheme screened the microorganisms in the calcareous sand. Making urease-producing microorganisms the dominant species improved the urease-producing ability and total urease activity; ultimately, the mineralization rate of calcareous sands could be increased. The level of change in the microbial community was a strong basis for the successful reinforcement of calcareous sand by stimulating native urease-producing microorganisms.

## Figures and Tables

**Table 1:** Particle parameters of calcareous sand.

*d*_10_	*d*_30_	*d*_60_	*C_u_*	*C_c_*
0.281 mm	0.385 mm	0.592 mm	2.107	0.891

**Table 2:** Factors and levels of the PB test.

Factor	Level	
	−1	+1
Molasses (g/L)	3.5	5.2
Sodium acetate (mM)	42.5	63.75
Corn steep liquor (g/L)	5	7.5
Soybean meal (g/L)	5	7.5
Ammonium sulfate (mM)	35	53
Ammonium chloride (mM)	100	151
Nickel chloride (mM)	0.01	0.015
Urea (mM)	333	500
YE (g/L)	0.2	0.3
pH	7.0	9.0

**Table 3:** Urease activity results from the PB tests.

Number	A	B	C	D	E	F	G	H	I	J	UA
1	1	−1	1	−1	−1	−1	1	1	1	−1	1.19
2	1	1	−1	1	−1	−1	−1	1	1	1	3.06
3	−1	1	1	−1	1	−1	−1	−1	1	1	4.43
4	1	−1	1	1	−1	1	−1	−1	−1	1	4.93
5	1	1	−1	1	1	−1	1	−1	−1	−1	0.35
6	1	1	1	−1	1	1	−1	1	−1	−1	1.46
7	−1	1	1	1	−1	1	1	−1	1	−1	2.29
8	−1	−1	1	1	1	−1	1	1	−1	1	2.64
9	−1	−1	−1	1	1	1	−1	1	1	−1	2.36
10	1	−1	−1	−1	1	1	1	−1	1	1	5.90
11	−1	1	−1	−1	−1	1	1	1	−1	1	3.68
12	−1	−1	−1	−1	−1	−1	−1	−1	−1	−1	1.39

Note: The variables A, B, C, D, E, F, G, H, I, and J represent molasses, sodium acetate, corn steep liquor, soybean meal, ammonium sulfate, ammonium chloride, nickel chloride, urea, YE, and pH, respectively.

**Table 4:** Results of PB design.

Item	Effect	*p* Value	Estimated Coefficient
Molasses	0.0167	0.705	0.0083
Sodium acetate	−0.5233	0.040	−0.2617
Corn steep liquor	0.0333	0.500	0.0167
Soybean meal	−0.4033	0.052	−0.2017
Ammonium sulfate	0.1000	0.205	0.0500
Ammonium chloride	1.2600	0.017	0.6300
Nickel chloride	−0.2633	0.080	−0.1317
Urea	−0.8167	0.026	−0.4083
YE	0.7967	0.027	0.3983
pH	2.6000	0.008	1.3000
Model		0.026	
R^2^ > 99%			

**Table 5:** Factors and levels of the CCD test.

Factor	Level				
	−2	−1	0	1	2
Sodium acetate (mM)	21.25	31.88	42.5	53.13	63.75
Ammonium chloride (mM)	100	120	140	160	180
YE (g/L)	0.1	0.2	0.3	0.4	0.5
pH	8	8.5	9	9.5	10

**Table 6:** Analysis of variance of the response surface model.

Source	df	Sum of Squares	Mean Square	*F* Value	*p* Value Prob > *F*
Model	14	34.7283	2.4806	23.63	<0.0001
A	1	0.0012	0.0012	0.01	0.917
B	1	0.4085	0.4085	3.89	0.066
C	1	5.1328	5.1328	48.90	<0.0001
D	1	0.2707	0.2707	2.58	0.128
A^2^	1	5.1562	5.1562	49.12	<0.0001
A^2^	1	10.6015	10.6015	101.00	<0.0001
C^2^	1	2.5502	2.5502	24.30	<0.0001
D^2^	1	11.0546	11.0546	105.32	<0.0001
AB	1	0.0814	0.0814	0.78	0.392
AC	1	0.3460	0.3460	3.30	0.088
AD	1	0.1442	0.1442	1.37	0.258
BC	1	2.4704	5.4704	23.54	<0.0001
BD	1	1.5234	1.5234	14.51	0.0023
CD	1	1.3439	1.3439	12.80	0.003
Misfit term					0.244
Pure error	6	0.4198			
Cor total	30	36.4077			
*R*^2^ = 95.39%		*R*^2^(adj.) = 91.35%			

Note: A, B, and C represent the concentrations of sodium acetate, ammonium chloride, and YE, respectively; D represents the pH.

## Data Availability

Data are not publicly available, although the data may be made available on request from the corresponding author.

## References

[1] Zhu C., Chen H., Meng Q., Wang R. (2014). Microscopic characterization of intra-pore structures of calcareous sands. Rock Soil Mech..

[2] Zhang Q., Ye W., Liu Z., Wang Q., Chen Y. (2022). Advances in soil cementation by biologically induced calcium carbonate precipitation. Rock Soil Mech..

[3] Whiffin V.S., van Paassen L.A., Harkes M.P. (2007). Microbial carbonate precipitation as a soil improvement technique. Geomicrobiol. J..

[4] Burbank M.B., Weaver T.J., Green T.L., Williams B.C., Crawford R.L. (2011). Precipitation of Calcite by Indigenous Microorganisms to Strengthen Liquefiable Soils. Geomicrobiol. J..

[5] Cheng L., Cord-Ruwisch R., Shahin M.A. (2013). Cementation of sand soil by microbially induced calcite precipitation at various degrees of saturation. Can. Geotech. J..

[6] DeJong J.T., Fritzges M.B., Nusslein K. (2006). Microbially induced cementation to control sand response to undrained shear. J. Geotech. Geoenvironmental Eng..

[7] Liu H., Xiao P., Xiao Y., Wang J., Chen Y., Chu J. (2018). Dynamic behaviors of MICP-treated calcareous sand in cyclic tests. Chin. J. Geotech. Eng..

[8] Zhang X., Chen Y., Zhang Z., Ding X., Xu S., Liu H., Wang Z. (2020). Performance evaluation of liquefaction resistance of a MICP-treated calcareous sandy foundation using shake table tests. Chin. J. Geotech. Eng..

[9] Jiang N.J., Yoshioka H., Yamamoto K., Soga K. (2016). Ureolytic activities of a urease-producing bacterium and purified urease enzyme in the anoxic condition: Implication for subseafloor sand production control by microbially induced carbonate precipitation (MICP). Ecol. Eng..

[10] Kirkland C.M., Thane A., Hiebert R., Hyatt R., Kirksey J., Cunningham A.B., Phillips A.J. (2020). Addressing wellbore integrity and thief zone permeability using microbially-induced calcium carbonate precipitation (MICP): A field demonstration. J. Pet. Sci. Eng..

[11] Wu J., Wang X.B., Wang H.F., Zeng R.J. (2017). Microbially induced calcium carbonate precipitation driven by ureolysis to enhance oil recovery. RSC Adv..

[12] Liu S., Dong B., Yu J., Cai Y. (2021). Evaluation of Biostimulation Efficacy on the Reinforcement of Calcareous Sand. J. Test. Eval..

[13] San Pablo A.C.M., Lee M., Graddy C.M.R., Kolbus C.M., Khan M., Zamani A., Nelson D.C. (2020). Meter-Scale Biocementation Experiments to Advance Process Control and Reduce Impacts: Examining Spatial Control, Ammonium By-Product Removal, and Chemical Reductions. J. Geotech. Geoenvironmental Eng..

[14] Gomez M.G., Anderson C.M., Graddy C.M.R., DeJong J.T., Nelson D.C., Ginn T.R. (2017). Large-Scale Comparison of Bioaugmentation and Biostimulation Approaches for Biocementation of Sands. J. Geotech. Geoenviron. Eng..

[15] Mahanty B., Kim S., Kim C.G. (2012). Assessment of a Biostimulated or Bioaugmented Calcification System with Bacillus pasteurii in a Simulated Soil Environment. Microb. Ecol..

[16] Islam M.T., Chittoori B.C.S., Burbank M. (2020). Evaluating the Applicability of Biostimulated Calcium Carbonate Precipitation to Stabilize Clayey Soils. J. Mater. Civ. Eng..

[17] Raveh-Amit H., Tsesarsky M. (2020). Biostimulation in Desert Soils for Microbial-Induced Calcite Precipitation. Appl. Sci..

[18] Dhami N.K., Alsubhi W.R., Watkin E., Mukherjee A. (2017). Bacterial Community Dynamicsand Biocement Formationduring Stimulation and Augmentation: Implications for Soil Consolidaton. Front. Microbiol..

[19] Gomez M.G., Anderson C.M., Dejong J.T., Nelson D.C., Lau X.H. Stimulating In Situ Soil Bacteria for Bio-Cementation of Sands. Proceedings of the Geo-Congress 2014.

[20] Gomez M.G., Graddy C.M.R., DeJong J.T., Nelson D.C., Tsesarsky M. (2018). Stimulation of Native Microorganisms for Biocementation in Samples Recovered from Field-Scale Treatment Depths. J. Geotech. Geoenviron. Eng..

[21] Kiasari M.A., Pakbaz M.S., Ghezelbash G.R. (2019). Comparison of Effects of Different Nutrients on Stimulating Indigenous Soil Bacteria for Biocementation. J. Mater. Civ. Eng..

[22] Gat D., Ronen Z., Tsesarsky M. (2016). Soil Bacteria Population Dynamics Following Stimulation for Ureolytic Microbial-Induced CaCO3 Precipitation. Environ. Sci. Technol..

[23] Wang Y.J., Han X.L., Jiang N.J., Wang J., Feng J. (2020). The effect of enrichment media on the stimulation of native ureolytic bacteria in calcareous sand. Int. J. Environ. Sci. Technol..

[24] Ministry of Water Resources of the People’s Republic of China (WMR) (1999). Specification of Soil Test, SL 237-1999.

[25] Zarastvand M.R., Asadijafari M.H., Talebitooti R. (2022). Acoustic wave transmission characteristics of stiffened composite shell systems with double curvature. Compos. Struct..

[26] Whiffin V.S. (2004). Microbial CaCO3 Precipitation for the Production of Biocement. Ph.D. Thesis.

[27] Deng X., Zhou H., Qu X.N., Long J., Peng P.Q., Hou H.B., Li K.L., Zhang P., Liao B.H. (2017). Optimization of Cd(II) removal from aqueous solution with modified corn straw biochar using Plackett-Burman design and response surface methodology. Desalination Water Treat..

[28] Miao L., Wu L., Sun X., Li X., Zhang J. (2020). Method for solidifying desert sands with enzyme-catalysed mineralization. Land Degrad. Dev..

[29] Chu J., Ivanov V., Naeimi M., Stabnikov V., Liu H.L. (2013). Optimization of calcium-based bioclogging and biocementation of sand. Acta Geotech..

[30] Okyay T.O., Rodrigues D.F. (2014). Optimized carbonate micro-particle production by *Sporosarcina pasteurii* using response surface methodology. Ecol. Eng..

[31] Al-Salloum Y., Abbas H., Sheikh Q.I., Hadi S., Alsayed S., Almusallam T. (2017). Effect of some biotic factors on microbially-induced calcite precipitation in cement mortar. Saudi J. Biol. Sci..

[32] Lin H., Suleiman M.T., Brown D.G. (2020). Investigation of pore-scale CaCO3 distributions and their effects on stiffness and permeability of sands treated by microbially induced carbonate precipitation (MICP). Soils Found..

[33] Al Qabany A., Soga K. (2013). Effect of chemical treatment used in MICP on engineering properties of cemented soils. Geotechnique.

[34] Li B. (2015). Geotechnical Properties of Biocement Treated Sand and Clay. Ph.D. Thesis.

[35] Stabnikov V., Jian C., Ivanov V., Li Y. (2013). Halotolerant, alkaliphilic urease-producing bacteria from different climate zones and their application for biocementation of sand. World J. Microbiol. Biotechnol..

[36] Ahenkorah I., Rahman M.M., Karim M.R., Teasdale P.R. Optimization of Enzyme Induced Carbonate Precipitation (EICP) as a Ground Improvement Technique. Proceedings of the Geo-Congress 2020.

[37] Van Paassen L.A., Daza C.M., Staal M., Sorokin D.Y., van der Zon W., van Loosdrecht M.C.M. (2010). Potential soil reinforcement by biological denitrification. Ecol. Eng..

[38] Van Paassen L.A., Harkes M.P., Van Zwieten G.A., Van Der Zon W.H., Van Der Star W.R.L., Van Loosdrecht M.C.M. Scale up of BioGrout: A biological ground reinforcement method. Proceedings of the 17th International Conference on Soil Mechanics and Geotechnical Engineering: The Academia and Practice of Geotechnical Engineering.

[39] Liu H., Xiao P., Xiao Y., Chu J. (2019). State-of-the-art review of biogeotechnology and its engineering applications. J. Civ. Environ. Eng..

[40] Gu Z., Chen Q., Wang L., Niu S., Zheng J., Yang M., Yan Y. (2022). Morphological Changes of Calcium Carbonate and Mechanical Properties of Samples during Microbially Induced Carbonate Precipitation (MICP). Materials.

[41] Yang Y., Li M., Tao X., Zhang S., He J., Zhu L., Wen K. (2022). The Effect of Nucleating Agents on Enzyme-Induced Carbonate Precipitation and Corresponding Microscopic Mechanisms. Materials.

[42] DeJong J.T., Mortensen B.M., Martinez B.C., Nelson D.C. (2010). Bio-mediated soil improvement. Ecol. Eng..

[43] Rowshanbakht K., Khamehchiyan M., Sajedi R.H., Nikudel M.R. (2016). Effect of injected bacterial suspension volume and relative density on carbonate precipitation resulting from microbial treatment. Ecol. Eng..

[44] Wang L., Liu S. (2021). Mechanism of Sand Cementation with an Efficient Method of Microbial-Induced Calcite Precipitation. Materials.

[45] Liu S., Yu J., Zeng W., Peng X., Cai Y., Tu B. (2020). Repair effect of tabia cracks with microbially induced carbonate precipitation. Chin. J. Rock Mech. Eng..

[46] Chittoori B.C.S., Pathak A., Burbank M., Islam M.T. (2020). Application of Bio-Stimulated Calcite Precipitation to Stabilize Expansive Soils: Field Trials. Geo-Congress.

[47] Dhami N.K., Mukherjee A., Reddy M.S. (2016). Micrographical, minerological and nano -mechanical characterisation of microbial carbonates from urease and carbonic anhydrase producing bacteria. Ecol. Eng..

[48] Bai Y., Chang Y., Liang J., Chen C., Qu J. (2016). Treatment of groundwater containing Mn(II), Fe(II), As(III) and Sb(III) by bioaugmented quartz-sand filters. Water Res..

[49] Park S.C., Baik K.S., Kim M.S., Chun J., Seong C.N. (2008). Nocardioides dokdonensis sp nov., an actinomycete isolated from sand sediment. Int. J. Syst. Evol. Microbiol..

